# A Tubular Electrochemical Reactor for Slurry Electrodes

**DOI:** 10.1002/celc.202000616

**Published:** 2020-06-25

**Authors:** Korcan Percin, Oliver Zoellner, Deniz Rall, Matthias Wessling

**Affiliations:** ^1^ DWI-Leibniz Institute for Interactive Materials Forckenbeckstr. 50 52074 Aachen Germany; ^2^ RWTH Aachen University Aachener Verfahrenstechnik-Chemical Process Engineering, Forckenbeckstr. 51 52074 Aachen Germany

**Keywords:** tubular, redox flow battery, vanadium, slurry electrode, conductive static mixer

## Abstract

The research on electrochemical reactors is mostly limited to planarly designed modules. In this study, we compare a tubular and a planar electrochemical reactor for the utilization of the slurry electrodes. Cylindrical formed geometries demonstrate a higher surface‐to‐volume ratio, which may be favorable in terms of current density and volumetric power density. A tubular shaped electrochemical reactor is designed with conductive static mixers to promote the slurry particle mixing, and the vanadium redox flow battery is selected as a showcase application. The new tubular design presents similar cell resistances to the previously designed planar battery and shows increased discharge polarization behavior up to 100 mA cm^−2^. The volumetric power density reaches up to 30 mW cm^−3^, which is two times higher than that of the planar one. The battery performance is further investigated and 85 % coulombic, 70 % voltage and 60 % energy efficiency is found at 15 mA cm^−2^ with 15 wt.% slurry content.

## Graphical abstract

**Geometry matters**: We designed a tubular shaped electrochemical reactor for slurry electrodes. The tubular reactor is used in a vanadium redox flow system as a showcase study. The tubular reactor achieves higher power density in comparison to a standard planar electrochemical reactor.

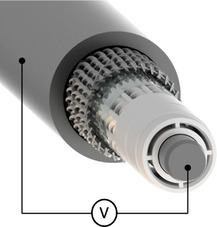

## 1 Introduction

Recent concerns about the environmental impact and sustainability of fossil‐fuel based energy sources have accelerated the research on renewable forms of energy. Fluctuations caused by these sources remain problematic and need to be handled before renewables can be efficiently utilized in the current electricity grid.^1^, ^2^ A promising way of overcoming this challenge is to use electrochemical storage and conversion processes, which are energy‐efficient, effective, and clean.^3^, ^4^, ^5^ However, further studies on electrochemical reactors are needed to improve their selectivity and efficiency.

All electrochemical reactors require two electrodes and an electrolyte for redox reactions to take place. Electrode materials and properties are one of the most researched components of electrochemical reactors. Usually, solid porous electrodes are used as high‐surface‐area electrode materials.^6^, ^7^, ^8^ Nonetheless, these electrodes still need improvement regarding their surface area as well as flexibility and kinetic behaviors.

Slurry electrodes, also known as flow electrodes, have been recently considered to be a dynamic replacement for solid porous electrodes in electrochemical systems.^9^, ^10^, ^11^, ^12^, ^13^ The scalability of conventional electrochemical reactors is mainly limited by the electrodes being not scaled up energy efficiently and cost‐effectively.^14^ By using conductive particle networks, a flowable electrolyte‐electrode dispersion can be maintained, which can be scaled up or down independently of reactor size. In this context, slurry electrodes enable a suitable environment for electrochemical processes, where the reactor design can be altered according to the electrode, gasket, or spacer requirements. Moreover, slurry electrodes are easy to produce and recyclable via a simple post‐process.^9^ Some applications have already proven to be suitable for slurry electrodes, such as flow‐electrode capacitive deionization systems,^15^ electrochemical flow capacitors,^16^ redox flow batteries,^17^ and semi‐solid lithium‐ion batteries.^18^ Commonly, solid porous electrodes are employed in these systems, but these electrodes perform best when pressed tightly to the current collectors to achieve the highest possible conductivity.^19^ In contrast to porous electrodes, no compression is necessary for the usage of the slurry electrodes in the cell. Instead, conductive static mixers can simply enable a sufficient activity for the slurry electrodes.^20^ This facilitates an easier realization of cell geometries diverging from the usual planar designs. Yet, there are no attempts to investigate different cell geometries for the slurry electrode applications.

One of the particular geometry that has been investigated for traditional electrochemical reactors is the cylindrical geometry. Some tubular PEMFC studies suggest better polarization performance at higher overpotential regions than classical planar designs.^21^, ^22^ Solid oxide fuel cells are also another choice of application for tubular structured modules, mostly because of their superior thermal stability.^23^, ^24^, ^25^ There are several studies on tubular‐shaped direct methanol fuel cells, where a better weight‐to‐volume ratio was hypothesized.^26^, ^27^, ^28^ Finally, Ressel et al. introduced a tubular module design for a traditional vanadium redox flow battery (VRB), which showed up to 70 mA/cm^−2^ polarization behavior. The study mentions 12 times higher area‐specific cell resistance (ASR), which is proposed to be caused by the lack of compression on the graphite felt electrodes.^29^

The effect of compression on the graphite felts have also been studied widely.^19^, ^30^, ^31^ These studies indicate that a higher degree of compression yields lower ASR values, which is favorable for the energy efficiency of the battery. However, the porosity of the felt is hindered by this compression, which causes higher pressure drops for the electrolyte flow and accordingly results in lower round trip efficiency for the system. This trade‐off has been concluded to be most efficient in the range of 20–40 % of felt compression in the VRB systems. Realizing sufficient compression in a tubular system is more challenging than in a planar system due to geometrical limitations. Therefore, higher cell resistances in tubular systems are expected when felt electrodes are used. On the contrary, slurry electrodes are promising for use in the tubular module design, since compression is not necessary.

**Figure 1:**
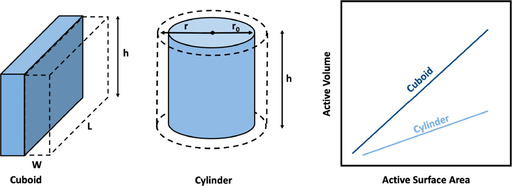
Cuboid and cylinder active surface area (left) and volume increase by active surface area graph (right).

An advantage of the tubular electrochemical systems is that cylindrical geometry enables a higher surface‐to‐volume ratio than planar systems. This can be proven if a similar active electrode surface area is chosen and the electrolyte volumes are compared. For instance, Figure 1 presents a cuboid with a plane active electrode surface area ($h{}^{*}L$ ) and cuboidal electrolyte volume flow (h×L×W ). Secondly, a cylinder with a circular active electrode surface area ($2\times \pi \times h\times r_{0}$ ) and cylindrical electrolyte volume flow ($\pi \times h\times (r^{2}-r_{0}^{2})$ ). The surface area increase in the cylindrical geometry has a lesser gain on the volume compared to the cuboid geometry (Figure 1). In another saying, if the surface area of both geometries is scaled up, the volume increase of the cylindrical shape would be smaller. Further, a smaller volume per surface area of tubular shape would be advantageous in terms of power density of the electrochemical reactor. Therefore, having a cylindrical reactor for an electrochemical process may be more versatile in terms of scaling up the modules without increasing the volume as exceedingly as it is for the planar systems. Thus, volumetric power density (power per volume) of the systems can be improved.

In this study, we discuss if a tubular geometry may be beneficial in terms of electrochemical activity for electrochemical reactors. The activity increase is expected because of the higher packing density per volume that can be achieved by tubular geometries. Therefore, a design and comparison of a lab‐scale tubular electrochemical reactor to a planar one is presented in this research. Previously, we established a slurry VRB combined with the static mixers which enable the usage of slurry electrodes.^20^ Slurry electrodes are suitable candidates for transforming the planar cell designs to tubular ones, due to the limitations in the standard solid electrodes. Herein, the tubular reactor is designed for the slurry vanadium system including conductive static mixers, and the battery performance is evaluated in terms of battery characteristics. Furthermore, volumetric power density evaluation is discussed by comparing the results to the previous planar slurry VRB.

## Experimental Section

### Materials

The current collectors of the module are made of impregnated graphite (Müller & Rössner GmbH, Germany), machined as a hollow cylinder (28 mm inner diameter, 3 mm wall thickness) and a rod (9 mm diameter). A Nafion^TM^ Tubing from PermaPure LLC is used as cation exchange membrane (17 mm inner diameter, 100 μm thickness). All supporting structures and static mixers are created by rapid polymer prototyping (Stratasys, Objet Eden 260 V). RGD525 is chosen for 3D‐print material as it provides sufficient mechanical and chemical durability. Static mixers were made conductive by firstly etching in 75 % sulfuric acid for 10 min and followed by spray coating with a conductive lacquer (Cramolin) for about ten layers. A conductive adhesive, Leit‐C (Sigma‐Aldrich Chemie GmbH), is used to decrease the contact resistances between static mixers and current collectors. Several different O‐ring sealings (EPDM, Landefeld Druckluft und Hydraulik GmbH) were placed in the assembly to prevent liquid leakages.

VRB experiments are conducted with commercially available vanadium electrolyte (GFE‐AMG Titanium Alloys & Coatings) consisting of 0.8 M V(III), 0.8 M V(IV) in total 4.5 M sulfate concentration. Slurry electrodes are prepared by dispersing graphite powder (synthetic, 20 μm, 20 m^2^g^−1^, Sigma‐Aldrich Chemie GmbH) into the electrolyte.

### Cell Design

**Figure 2:**
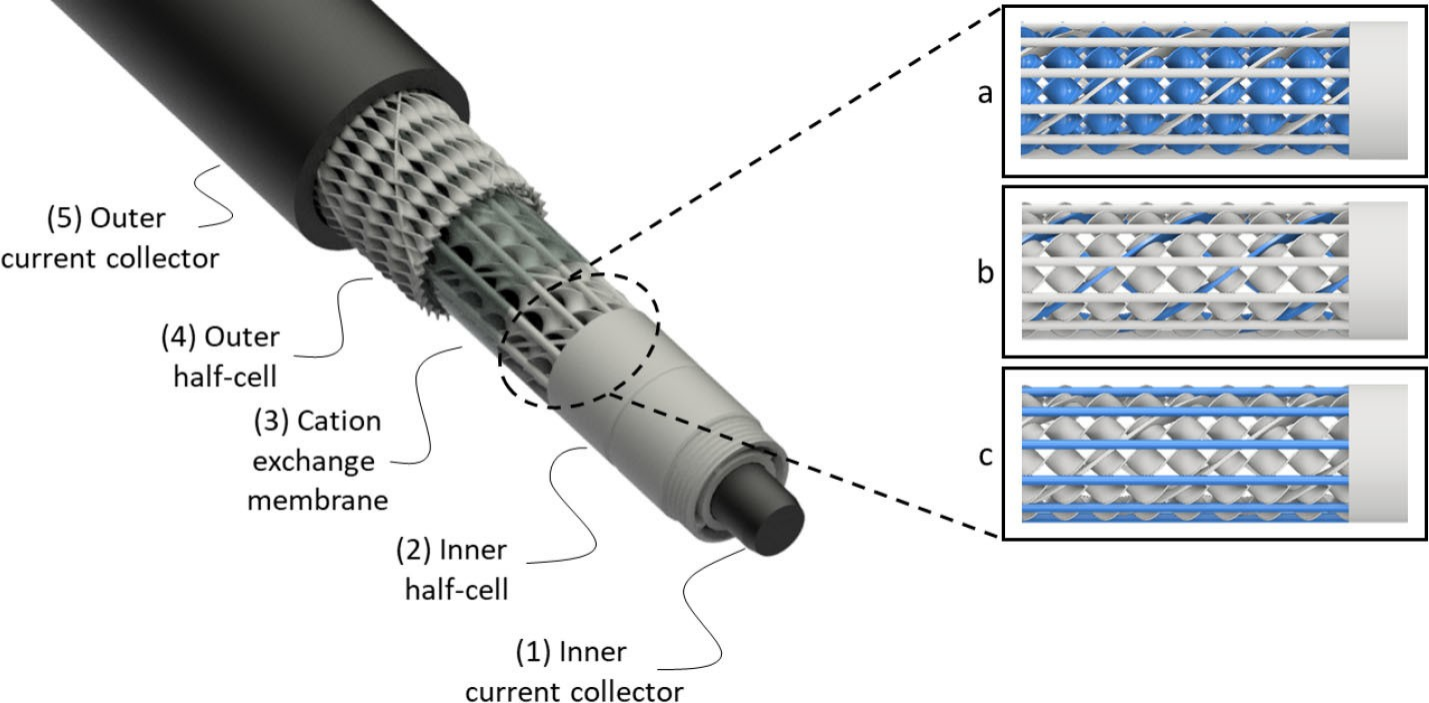
Design layers of the tubular assembly with spot in the inner halfcell structure; a) single‐twisted static mixers, b) global mixers, c) support columns.

The main layers of the tubular redox flow battery can be seen from Figure 2. The tubular design consists of an inner and an outer half‐cell separated by a tubular cation exchange membrane. Both half‐cells include conductive static mixers as flow distributors for slurry electrodes. A rod shaped current collector (1) contacts the inner half‐cell and placed at the very center of the design. The active geometrical surface area of the rod is defined as 17.5 cm^2^. The inner half‐cell (2) consists of static mixers in the core that forms the active inner volume. It also comprises inlet and outlet for the inner electrolyte flow. The flat tubular surfaces on both edges serve for membrane fastening with the help of O‐rings. The central part of the static mixer is consisting of (a) single‐twisted static mixers adapted from Fritzmann et al.,^32^ (b) global mixers, (c) support columns for mechanical stability. The whole part is pushed over the current collector rod (1) and the electrical conductivity is improved by applying the conductive adhesive. A cylindrical current collector (5) is positioned at the outer shell of the module and contacts the outer half‐cell (4). The outer half‐cell static mixers are made of only the single‐twisted static mixers (a) and the global mixers (b). It is also linked with the conductive adhesive to the outer current collector that radially encloses the outer half‐cell.

**Figure 3:**
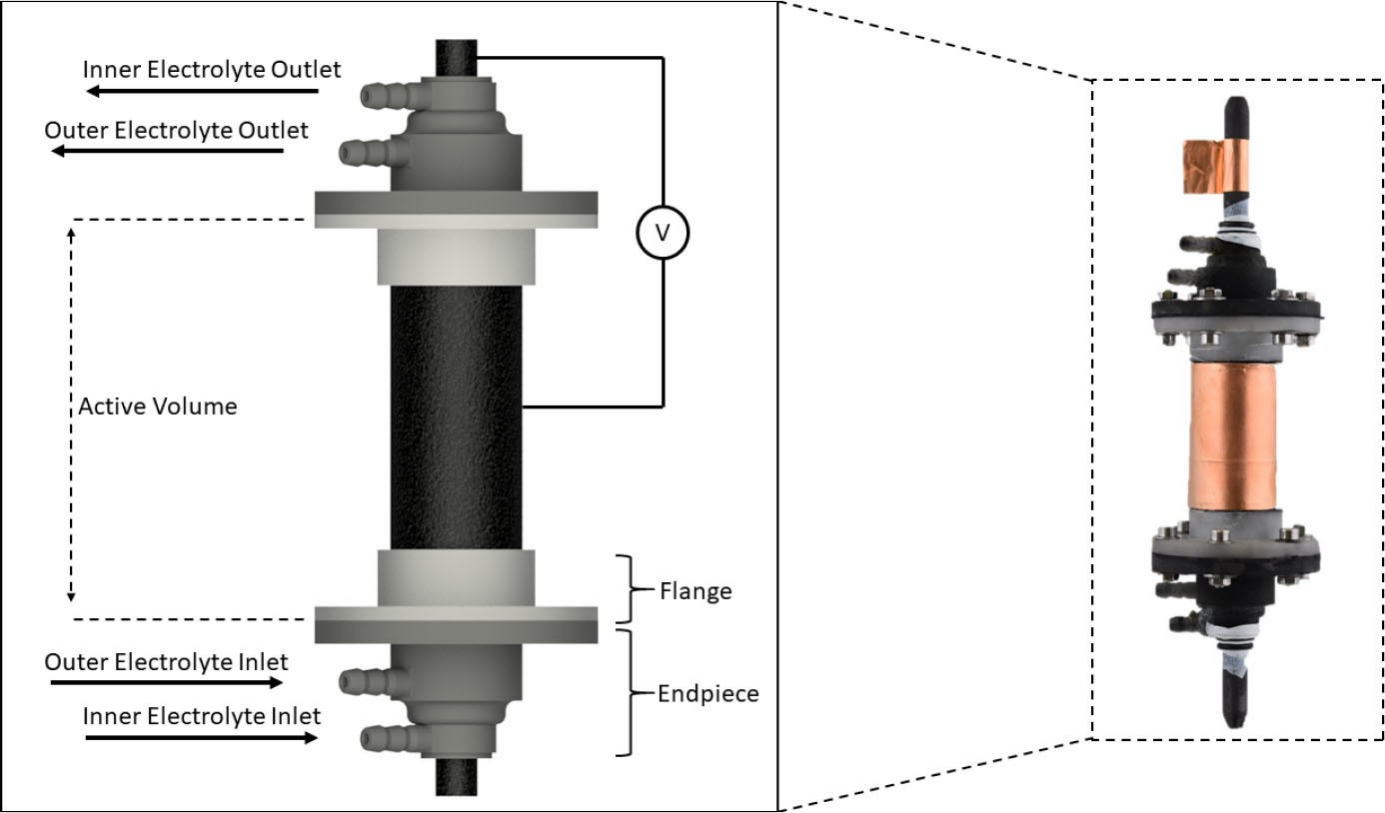
Complete tubular module illustration (left) and realization (right).

The overall illustration and the completed module are presented in Figure 3. The design is finalized with the end pieces on both sides of the module. The end pieces distribute the outer and inner electrolyte to the static mixers while preventing leakage from the half‐cells. Two flanges on the outer current collector connect both end pieces to the module and fighten the module through screws. Thread seal tape (PTFE) is used throughout the assembly to prevent the leakages. Finally, conductive copper tapes are rounded on the outer and the inner current collector for the connection to the power supplies.

### Electrochemical Measurements

All experiments are carried out with the novel designed tubular module. Glass vessels are used for each electrolyte with outlets at the bottom and inlets on the upper sides. Vessels are purged with constant $N_{2}$ feed to avoid air oxidation of vanadium species. An electrolyte flow rate of 20 ml min^−1^ is maintained by a peristaltic pump (Masterflex L/S, Cole Palmer) and each electrolyte vessel is filled with 50 ml electrolyte. Slurry electrodes are prepared by dispersing graphite powder with weight ratios of 5, 10, and 15 % into vanadium electrolytes and stirring until a well dispersed mixture is maintained.

A potentiostat/galvanostat is used with an impedance module (PGSTAT302 N, Metrohm GmbH) for the electrochemical measurements. Three different types of measurements were conducted, namely electrochemical impedance spectroscopy (EIS), polarization analysis, and charge‐discharge experiments. Firstly, the electrolytes are charged to 50 % state of charge (SOC) by using a previously built standard VRB. Then, EIS and polarization experiments are conducted, respectively for each slurry content with 0 (only electrolyte) to 15 wt.–%. The EIS measurements are done in the frequency region of 100 kHz to 100 mHz at a sine wave signal amplitude of 10 mV. The ASR is calculated from high frequency EIS data to calculate ohmic drop corrected potential values for the polarization study. EIS measurements are followed by the polarization study, where a series of galvanostatic charge and discharge steps are performed. Constant current (CC) is applied for 30 s and corresponding cell potential is recorded with cutoff voltage of 1.9 V for charge and 0.8 V for discharge. After each CC step, the open circuit potential (OCP) of the battery is brought to the initial OCP value, where the SOC is set to 50 %. Finally, charge‐discharge experiments are performed in the tubular module utilizing discharged 15 wt.–% slurry electrodes by applying CC of 15 mA cm^−2^. Consequently, energy and coulombic efficiencies are evaluated for each charge‐discharge cycle.

## 2 Results and Discussion

The tubular electrochemical module is designed to showcase a possible surface‐to‐volume ratio advantage against traditional planar‐designed electrochemical reactors. The slurry electrode VRB is chosen to demonstrate the capabilities of the tubular design and to incorporate the advantages of the dynamic feature of the slurry electrodes. The limiting active surface area of the tubular battery is built to be similar to our previously published static‐mixer installed planar VRB to moderate the surface area dependent inaccuracies.^20^ The limiting active surface area of the tubular battery is $17.5\;\;cm^{2}$ , while the planar battery's active surface area is $18\;cm^{2}$ . Thereby, all the results in the following section are presented in comparison to our previous planar VRB.

The tubular vanadium redox flow battery is firstly evaluated by the EIS measurements. The evaluation of the Nyquist plot is done for three basic resistances (1) ohmic resistances (R_Ω_) where the imaginary resistances are zero at high frequency region, (2) charge transfer resistances (R_ct_) are characterized at the mid‐range frequencies with a semi‐circle, followed by the (3) mass transport resistances (R_m_) at low frequencies with an inclining tail at the end of the nyquist plot.

**Figure 4:**
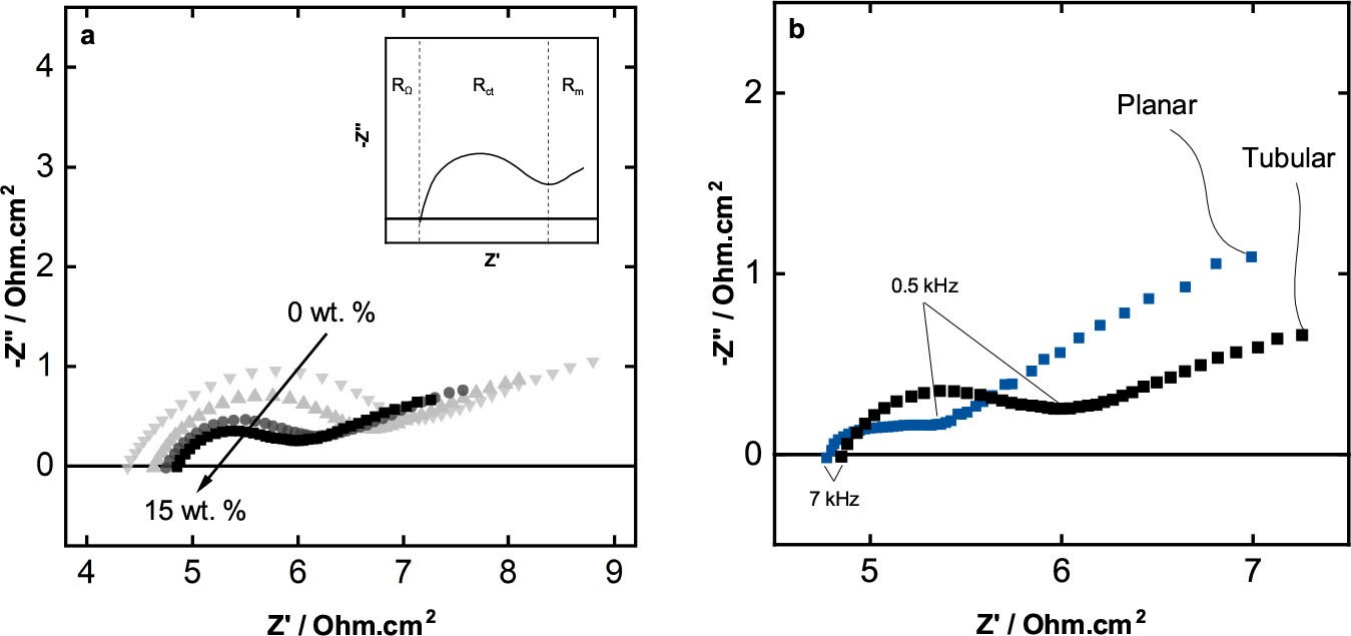
Nyquist plots of a) slurry concentration effect on the tubular module, a) 0 (▾), 5 (▴), 10 (•), 15 (▪) wt.% and b) comparison of planar to tubular batterywith each 15 wt.% slurry.

Figure 4a shows the Nyquist plots of the tubular slurry reactor with particle content from 0 wt.% to 15 wt.%. Ohmic resistance of the system increases gradually with the increase of the particle concentration. R_Ω_ of the 0 wt.% slurry is 4.4 Ωcm^2^ and increases up to 4.9 Ωcm^2^ for the 15 wt.%. The increase of ohmic resistance with increasing particle content has been also shown by Rommerskirchen et al. for the flow capacitive deionization systems where activated carbon particles used as active material. ^[33]^ This phenomen can be explained by the particles blocking the ionic transport in the cell. Higher particle content yields in a longer ionic pathway, thus higher ohmic resistance. Following the ohmic resistance, we observe a drastic decrease for the charge transfer resistances being significantly smaller with increasing slurry content. Overall charge transfer resistances decreased from 2.8 Ωcm^2^ (0 wt.%) to 1.1 Ωcm^2^ (15 wt.%). Lohaus et al. simulated a VRB slurry system, where a distinct correlation between particle concentration and particle percolation is found.^34^ Due to the particle percolation that develops in the slurry flow field, a greater charge transfer is expected from the current collectors to the particles. Therefore, we observe less R_ct_ while increasing the amount of the particle content in the electrolyte. The interpretation of increasing ohmic resistance and decreasing charge transfer resistance can be better understood, when the difference between the charge transfer through ions and particles are defined. The increasing amount of particles causes a low ionic conductivity due to blocked ionic pathways. In contrast, higher particle content improve the charge transfer from the current collectors to the particles, because more particles enable more electronic conductivity.^35^

Low frequency region directly after the half circles shows relatively low angled tail meaning less capacitive diffusion limitations. However, all the slurry concentrations show nearly identical low frequency behavior with only a shift by virtue of the changes in R_Ω_ and R_ct_. Therefore, we conclude that the diffusion limitations are not highly dependent on the particle content in the tubular reactor.

We choose 15 wt.% particle content as a comparison experiment to the previously designed planar slurry VRB and present EIS results for both 15 wt.% tubular and planar VRB in the Figure 4b. The planar module presents slightly lower R_Ω_ with a difference of approx. 0.1 Ωcm^2^, which may be caused by the difference in the electrolyte gap between both modules. The tubular design has a larger distance between the current collectors and membrane leading to a higher electrolyte gap, thus higher R_Ω_. However, the difference is negligible when we consider the total ohmic resistances. Surprisingly, R_ct_ of the planar module shows nearly three times lower resistance, while mass transport related resistances are comparable. It can be reasonably assumed according to these results that the planar module has less resistances, which would be the first indication for higher energy efficiencies. Furthermore, frequency regions for both tubular and planar setups show similar values being roughly 7 kHz for R_Ω_ and 0.5 kHz for the beginning of R_m_.

**Figure 5:**
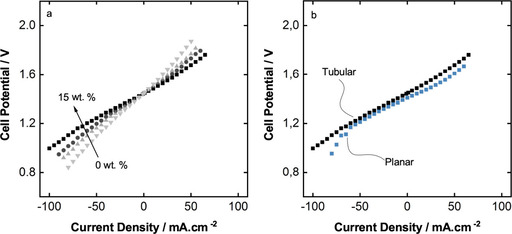
IR corrected cell polarization graphs a) 0 (▾), 5 (▴), 10 (•), 15 (▪) wt.% slurry content in the tubular cell, b) comparison of planar to tubular battery with each 15 wt.% slurry content.

Figure 5 shows the results of the polarization study on the tubular slurry VRB with IR corrected potential values. The measurements are all maintained at 50 % SOC and, JR correction is done according to the R_Ω_ values from Figure 5a. Polarization study is done by performing constant current charge and discharge on the battery and the voltage response of the system is recorded after it reaches a stable state. In such battery applications, it is desired to have the highest possible potentials during the discharge and lowest possible potential during the charging process. The potential cut‐off range is usually kept between 0.9 and 1.7 V for charge and discharge, respectively. The results from Figure 5a indicate a positive correlation between particle concentration and polarization behavior. 15 wt.% slurry shows the lowest overpotentials on both charge and discharge sides while reaching almost −100 mA cm^−2^ for discharging and 60 mA cm^−2^ for charging in the potential range of 0.8 to 1.8 V. Furthermore, the polarization curves become flattered with increasing content of the slurries, which is desired for the VRB application. In Figure 4b, we compare the polarization of the tubular and the planar module configurations consisting of 15 wt.% slurry. A difference can be detected on the discharge side, while the charge side is relatively similar. On the discharge side, the planar battery can only reach up to 75 mA cm^−2^. This difference can be explained with the better cell and static mixer design in the tubular module. The tubular module has a high packing density for the conductive static mixers. The conductive static mixers behave also as current collectors. Yet, the surface area of both modules is still calculated by the geometrical surface area of the graphite plate (planar) and graphite rod (tubular). By using more conductive static mixers, there is a larger contact surface available for the particles while the geometrical surface area is not changed. Consequently, higher current densities can be reached while the geometrical surface areas are similar. Secondly, as a result of having better mixing in the tubular module, the polarization at the high current region shows less steep voltage decrease. This region, where the high current density causes steep overpotential changes, is usually described by the mass transport related limitations.^36^

**Figure 6:**
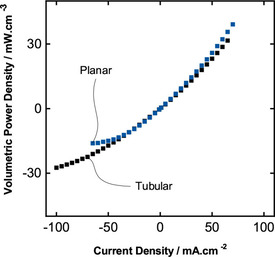
Volumetric power density comparison of planar and tubular battery with each 15 wt.% slurry content

One of the main reasons to strive for a tubular module design is the possible higher volumetric power density due to the improved surface‐to‐volume ratio of tubular structures. Therefore, the polarization data should be transformed into volumetric power density. However, both planar and tubular slurry VRBs are not fully optimized in terms of sealing and housing, which highly affects the total volume. Hence, we consider a real and equal basis for an objective comparison by reducing the volume to the core structure. The core of the planar cell is defined as outer bounds of graphite plates from each side, while the core of the tubular cell is defined as the outer diameter of the outer graphite tube. The comparison of the volumetric power density of tubular and planar modules for the 15 wt.% slurry electrodes can be seen from Figure 6. Higher discharge power density can be reached with the tubular design up to 30 mW cm^−3^. Additionally, for the planar design the power density reaches its plateau region at about 15 mW cm^−3^, while the tubular module does not indicate any plateau region yet for the discharge process. An insignificant difference on the charge side can be detected for the planar module showing about 5 mW cm 10^–3^ higher power density. Overall, we conclude a better performance for the tubular module because of the greater volumetric discharge power density, since we aimed to prove the advantage of the tubular structures due to their surface‐to‐volume ratio. Besides, the tubular module can be efficiently designed longer than current tubular modules resulting in a larger surface area without affecting the total volume as it would be for the planar module. In addition, an even higher increase in volumetric power density may be achieved.

**Figure 7:**
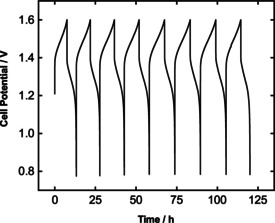
Cyclic charge and discharge for 15 wt.% slurry electrodes at 15 mA cm^−2^ constant current

Further, the battery performance of the tubular VRB is briefly studied by cyclic charge‐discharge testing with 15 wt.% slurry electrodes. An intermediate current density has been chosen concerning the polarization studies as 15 mA cm^−2^ for both charging and discharging. Figure 7 presents the cell potential among eight cycles. An ohmic overpotential drop approx. 0.25V can be detected after each charge cutoff. We reported similar results in our previous work, where we introduced the slurry electrodes for the VRB applications by using conductive static mixers.^20^ Furthermore, the ohmic resistances from EIS results in Figure 4b suggests a similar ASR for both planar and tubular modules. The ohmic overpotential is caused by the cell resistances that persists with the utilization of the slurry electrodes. We suggest that this can be improved by increasing the electrical conductivity of the static mixers. It is important to note that static mixers are produced by additive manufacturing to be able to create their complex geometry. The material for the static mixers is not conductive. Therefore, a graphite coating is applied on top of the polymer material, creating a conductive layer. The same procedure is applied for the tubular designed static mixers. However, the graphite coating is still relatively resistive (<100Ωcm ) resulting in higher contact resistances. Hence, improving the resistivity of these static mixers may reduce the overall ohmic losses.

**Figure 8:**
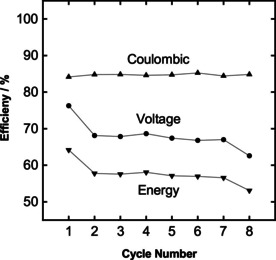
Tubular battery coulombic (▴), voltage (•), and energy (▾) efficiencies

The performance of the battery is further evaluated through coulombic (CE), energy (EE), and voltage (VE) efficiencies. Figure 8 shows the efficiencies over the cycles of charge and discharge. The CE represents the ratio of the charge amount during the charge and discharge. An average CE of 85 % is observed for the tubular VRB. Slightly low level of CE can be explained by the usage of the slurry electrodes. Recently, we proved that better mixing and conductivity of the static mixers provide better charge transfer to the particles, thus higher CE can be achieved.^20^ We believe that improving the contact resistances between the static mixers and the graphite current collectors may yield in desired results. Moreover, we introduced a novel way of static mixing using global mixers around the whole tubular structure. The mixing properties of these global mixers should be studied and validated in terms of the flow profile of the electrolyte and the particles. Undesired flow patterns might have been produced, due to the introduction of global mixing, causing low particle‐current collector interactions. The EE is the ratio of the consumption of the total energy for the charging process and the total energy released during the discharging process. The EE of the battery is found to be approx. 60 %, which is also relatively low compared to the literature values (75–90 %) for standard VRBs.^37^ On the other hand, it is similar to the findings of the tubular VRB made from standard graphite felt electrodes.^29^ Finally voltage efficiency is found to be around 70 % being slightly lower than literature values (80‐95 %), which is expected as a result of both lower CE and EE.^37^, ^38^, ^39^

## 3 Conclusion

In this research, a comparison of tubular and planar electrochemical reactors is studied using a slurry VRB as a showcase application. According to our previous findings, slurry electrodes can be utilized in a VRB by using conductive static mixers. Therefore, a tubular electrochemical reactor with 3D‐printed static mixers is designed to match the active current collector surface area with our previous planar design. Similar cell resistances are found through electrochemical impedance spectroscopy showing no extra ohmic or mass transport related resistances, making the comparison between tubular and planar modules easier. The polarization study of the tubular cell shows up to 100 mA cm^−2^ discharge current density while the planar battery can reach only up to 75 mA cm^−2^. Furthermore, when comparing the volumetric power densities of the two battery modules, almost twice as high volumetric power density of the two can be reached by using the tubular module, which results from higher surface‐to‐volume ratio. We state that, due to the surface‐to‐volume ratio of cylindrical forms, increasing the active surface area would not result in explicitly higher volumes as would be the case for a cuboid form. Therefore, scaling up the tubular electrochemical reactor would lead to an even higher volumetric power density. Even though this study focuses only on the electrochemical performance of such reactor designs and proves higher activity, the challenges manufacturing such systems persist. Further electrochemical processes should be considered that may benefit from the higher power densities likely to be delivered by tubular formed electrochemical reactors.

## Conflict of interest

The authors declare no conflict of interest.

## Supporting information

As a service to our authors and readers, this journal provides supporting information supplied by the authors. Such materials are peer reviewed and may be re‐organized for online delivery, but are not copy‐edited or typeset. Technical support issues arising from supporting information (other than missing files) should be addressed to the authors.

Supplementary
