# Supplementary material for: A Tubular Electrochemical Reactor for Slurry Electrodes
Source: ChemElectroChem. 2020 Jun 25;7(12):2665–71. doi: 10.1002/celc.202000616 (PMC7386916; doi:10.1002/celc.202000616)
Supplement: Supplementary file 1 — Supplementary [file CELC-7-2665-s001.pdf]

Author Contributions

K.P. Conceptualization:Lead; Data curation:Lead; Investigation:Lead; Methodology:Lead; Visualization:Lead; Writing - Original Draft:Lead; Writing - Review & Editing:Lead
O.Z. Conceptualization:Equal; Data curation:Equal; Investigation:Equal; Methodology:Equal
D.R. Conceptualization:Supporting; Writing - Review & Editing:Supporting
